# Automatic Modulation Recognition Based on a DCN-BiLSTM Network

**DOI:** 10.3390/s21051577

**Published:** 2021-02-24

**Authors:** Kai Liu, Wanjun Gao, Qinghua Huang

**Affiliations:** School of Communication and Information Engineering, Shanghai University, Shanghai 200444, China; WanJun0124@shu.edu.cn (W.G.); qinghua@shu.edu.cn (Q.H.)

**Keywords:** automatic modulation recognition, deep complex network, convolutional neural network, bidirectional long short-term memory network

## Abstract

Automatic modulation recognition (AMR) is a significant technology in noncooperative wireless communication systems. This paper proposes a deep complex network that cascades the bidirectional long short-term memory network (DCN-BiLSTM) for AMR. In view of the fact that the convolution operation of the traditional convolutional neural network (CNN) loses the partial phase information of the modulated signal, resulting in low recognition accuracy, we first apply a deep complex network (DCN) to extract the features of the modulated signal containing phase and amplitude information. Then, we cascade bidirectional long short-term memory (BiLSTM) layers to build a bidirectional long short-term memory model according to the extracted features. The BiLSTM layers can extract the contextual information of signals well and address the long-term dependence problems. Next, we feed the features into a fully connected layer. Finally, a softmax classifier is used to perform classification. Simulation experiments show that the performance of our proposed algorithm is better than that of other neural network recognition algorithms. When the signal-to-noise ratio (SNR) exceeds 4 dB, our model’s recognition rate for the 11 modulation signals can reach 90%.

## 1. Introduction

As a significant technology for noncooperative wireless communication systems, automatic modulation recognition (AMR) plays an important role in practical civil and military applications, such as cognitive radio, interference recognition and spectrum monitoring [1]. In the absence of prior knowledge, it can identify the modulation type of an intercepted signal, providing parameter information for subsequent demodulation [2].

Traditional AMR algorithms can be divided into two categories. One is based on maximum likelihood (ML) theory [3], and the other is a feature-based (FB) method [4]. The first approach uses probability theory, hypothesis test theory and an appropriate decision strategy to solve the AMR problem. The feature-based approach first extracts the modulated signal characteristics and then completes the recognition using classifiers. In feature-based methods, the number of selected features influences the recognition performance. The main features used for modulation signal identification include instantaneous amplitude, phase, frequency, high-order cumulant [5], cyclic spectrum [6] and wavelet characteristics [7]. Many of the classifiers used are based on machine learning algorithms; these include decision trees, support vector machines (SVMs) [8] and artificial neural networks (ANNs) [9].

In recent years, deep learning (DL), which is a powerful machine learning approach, has achieved great success in diverse fields such as image classification [10] and speech recognition [11]. The concept of DL comes from the research of ANNs. A multilayer perceptron with multiple hidden layers is a DL structure. DL forms a more abstract high-level representation attribute category or feature by combining low-level features to discover distributed feature representations of data. Its goal is to allow machines to have the ability to analyze and learn similar to humans and recognize data such as text, images and sounds. DL solves nonlinear classification problems by using activation functions and uses regularization to improve the robustness of the model [12]. The DL-based methods cascade multilayer nonlinear processing units to extract features. This approach can automatically optimize the extracted features to minimize later classification errors.

Many scholars have applied DL to the field of AMR in recent years. For example, Lee et al. [13] proposed a new method that calculated 28 statistical characteristics of five modulation signals; these were then sent to a fully connected feedforward network to conduct classification. Wang Yu at al. [14] trained CNN on samples composed of in-phase and quadrature component signals to distinguish modulation patterns that are relatively easy to identify. At the same time, a CNN based on the constellation map was designed to identify modulation modes that were difficult to distinguish in previous CNNs and improved the ability to classify QAM signals under low SNRs. Li et al. [12] studied the AMR method based on the original IQ signal under the parameter estimation error. First, the influence of parameter estimation error on the performance of CNN classifier is analyzed. Then, an AMR method based on spatial transformation network (STN) is proposed, which improves the robustness under parameter estimation errors. Li et al. [15] proposed a deep joint learning algorithm based on CNN and kernel collaborative representation and discriminative projection (KCRDP), including deep learning and kernel dictionary learning, which improves the adaptability of small samples and reduces the computational complexity without prior knowledge and feature enhancement processing.

In 2016, O’Shea et al. [16] generated a public dataset of modulated signals, named RML2016.10a, using GNU Radio software; then, they used a convolutional neural network (CNN) to identify the modulated signals. Subsequent studies have also adopted this dataset for AMR research. That same year, O’Shea [17] proposed a method to optimize the CNN structure. West et al. [18] applied a CNN, a residual network (Resnet), a convolutional long short-term memory deep neural network (CLDNN) and an Inception network to the modulated signal identification task and compared their respective recognition performances. The results show that the modulation signal recognition performance is not solely dependent on the network depth. Zhang et al. [19] proposed a preprocessing signal representation that combined the in-phase, quadrature and fourth-order statistics of the modulated signals. Omar S. Mossad et al. [20] proposed a CNN that used a multitask learning scheme (MTL-CNN) to reduce the confusion between similar classes. Kumar et al. [21] introduced a signal distortion correction module (CM) to estimate the carrier frequency offset and phase noise of the received signal to improve modulation recognition accuracy of deep learning schemes. Liu [22] proposed a group lasso based lightweight DNN for AMR which can learn to prune via automatically removing the neurons in hidden layers.

In 2020, Jakob et al. [23] used a linear combination to enable the DL architecture to perform complex convolutions (CVCs) and learn the characteristics of the real and imaginary parts of modulated signals. Wu et al. [24] constructed a CNN followed by a long short-term memory (LSTM) model as the classifier (CNN-LSTM) to efficiently explore the temporal and spatial correlation. However, to the best of our knowledge, none of the currently available methods fully consider the phase and contextual information of the modulated signals simultaneously.

In view of the current problems, this paper proposes a deep complex network that cascades bidirectional long short-term memory network (DCN-BiLSTM). First, deep complex network (DCN) layers with convolution kernels of different scales are connected; then, the bidirectional long short- term memory (BiLSTM) layers are cascaded. Finally, a softmax classifier is used to classify 11 kinds of digitally modulated signals. The main contributions of this paper are as follows:

(1) To the best of our knowledge, this study is the first to cascade DCN and BiLSTM models and apply them in the AMR field.
(2) We demonstrate the effectiveness of the DCN-BiLSTM network through experiments. The experimental results show that the performance of the proposed algorithm is better than that of other neural network recognition algorithms. When the signal-to-noise ratio (SNR) exceeds 4 dB, the recognition rate of our proposed model on the 11 modulation signals can reach 90%.

The remainder of this paper is organized as follows. Section 2 shows the signal model. Section 3 proposes the DCN-BiLSTM network model and introduces its components. In Section 4, we report the results of an experiment conducted to evaluate the proposed method and provide the optimal parameter configuration for the DCN-BiLSTM model. In addition, we use a cross-validation method to evaluate the network. Finally, Section 5 concludes this paper.

## 2. Signal Model

**Figure 1:**
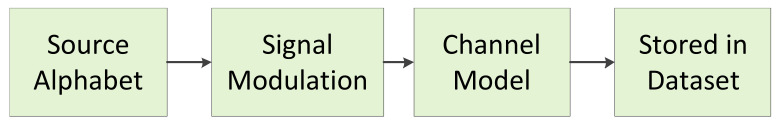
Data generation scheme in [16].

This paper uses the open dataset named RML2016.10a [16]. Figure 1 illustrates the dataset generation technique used in [16]. For the Rayleigh fading channel, the received signal can be expressed as

(1) $$r\left(n\right)=\sum_{l=1}^{L}h_{l}\left(n\right)s\left(n-n_{l}\right)e^{j2\pi f_{dl}nT_{0}}+\omega \left(n\right)$$

where s(n) is the modulated signal sent by the communication transmitter and *L* is the number of multipaths; $h_{l}\left(n\right)$ is the Rayleigh fading factor of the *l*th path; $n_{l}$ is the delay of the *l*th path; $f_{dl}$ represents the Doppler frequency; and ω(n) is additive white Gaussian noise. In addition, to ensure that the channel model is similar to a real channel, the channel model of this dataset includes the sampling rate and carrier rate offsets. The specific modulation types and parameters are shown in Table 1.

For a received signal, if we know the modulation type range, sampling frequency, sampling rate offset range, carrier rate offset range and the other signal parameters included in Table 1, we can use the recognition system model proposed in Section 3.

## 3. The Proposed Algorithm

### 3.1. DCN-BiLSTM Network Model

**Figure 2:**
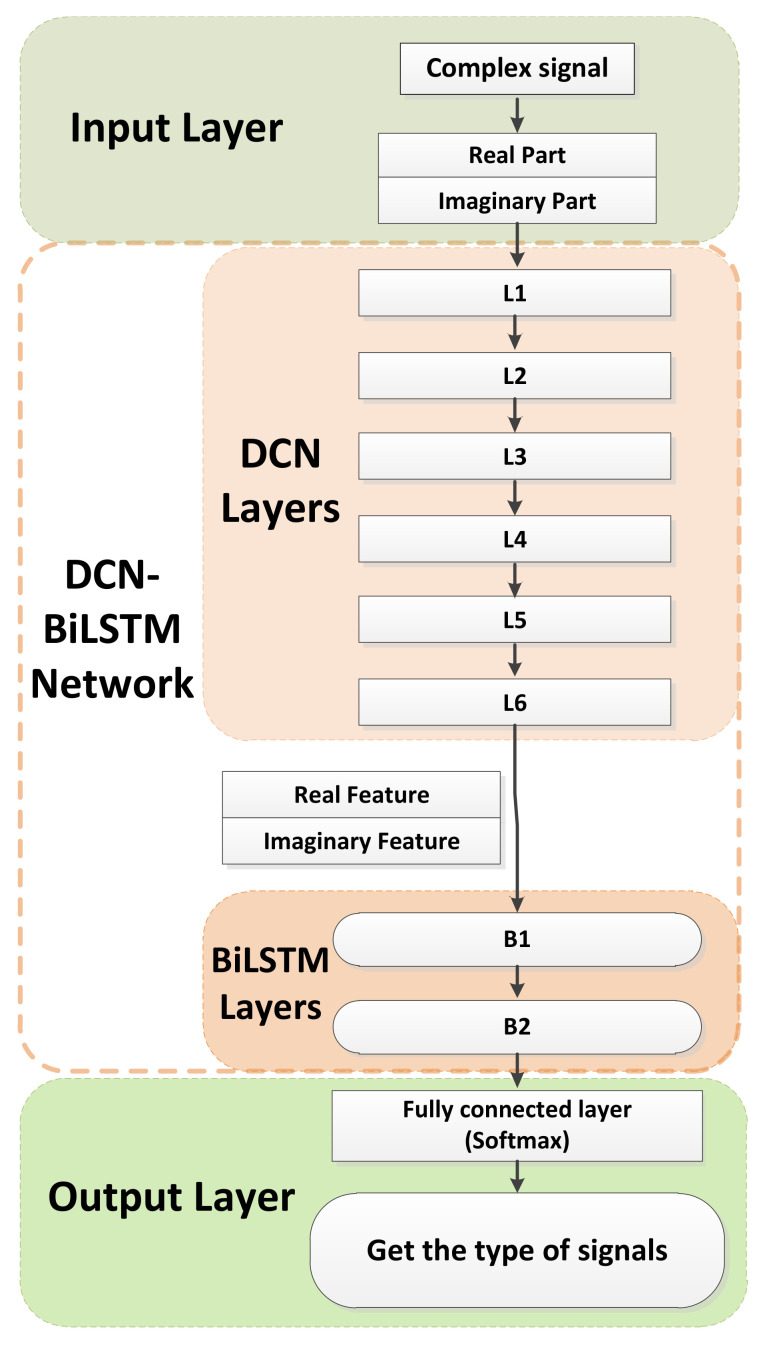
DCN-BiLSTM network identification system model.

As shown in Figure 2, we propose the DCN-BiLSTM network model for AMR. First, we preprocess the received signal and divide it into I and Q components. Then, we send I and Q to the DCN-BiLSTM network, which is designed for identification. Finally, we obtain the identified signal type. The network has four parts: an input layer, DCN layers, BiLSTM layers and a fully connected layer.

In the DCN layers, the I-channel data of the signal are convoluted with the I-channel convolution kernel of the complex-valued convolution kernel, while the Q-channel data are convoluted with the Q-channel convolution kernel. After convolution, the real features and imaginary features are output. The activation function for the complex-valued convolution is a rectified linear unit (ReLU) function, which is defined as follows:

(2) ReLU(x)=x,x>00,x≤0,

where *x* is the input. When x>0, the activation function has a linear relationship with the input.

The BiLSTM layers connect the contextual information among signals and build a bidirectional long short-term memory model for the extracted features. The fully connected layer uses the softmax activation function to output the predicted probability of the modulation information.

(3) $$y_{i}=S{\left(z\right)}_{i}=\frac{e^{z_{i}}}{\sum_{j=1}^{C}e^{z_{j}}},i=1,\ldots ,C,$$

where *z* is the output of the previous layer and eventually forms the input to the fully connected layer; *C* is the input dimension and the number of modulation types; and $y_{i}$ is the probability of an unknown signal being predicted as category *i*.

The algorithm uses the cross-entropy loss function to calculate the gradient in reverse to update the bias and weight values. The back-propagation update process is as follows

(4) $$\theta_{n+1}=\theta_{n}-\eta \frac{\partial \varphi (y,f(x,\theta ))}{\partial \theta_{n}},$$

where $\theta_{n}$ is the bias or weight of the last moment; η is the learning rate; and φ is the loss function.

### 3.2. Deep Complex-Valued Network Module (DCN)

The DCN [25] layers are composed of many complex-valued convolution kernels. These complex-valued convolution kernels of different scales are stacked together to perform a hierarchical convolution operation on the input signal.

In the complex-valued convolution operation, the real and imaginary parts are convolved separately. In Cartesian notation, the complex input matrix is defined as $M=M_{R}+iM_{{}_{I}}$. Similarly, the complex-valued convolution kernel matrix is defined as $K=K_{R}+iK_{I}$. These parameters, including $M_{R},M_{I},K_{R},K_{I}$, are all real-valued matrices. The complex-valued convolution expression is

(5) $$M^{{}^{\prime }}=M*K=\left(M_{R}+iM_{I}\right)*\left(K_{R}+iK_{I}\right).$$

where * is the operation of convolution. The above formula can be expanded to

(6) $$M^{\prime }=\left\{M_{R}*K_{R}-M_{I}*K_{I}\right\}+i\left\{M_{R}*K_{I}+M_{I}*K_{R}\right\}.$$

**Figure 3:**
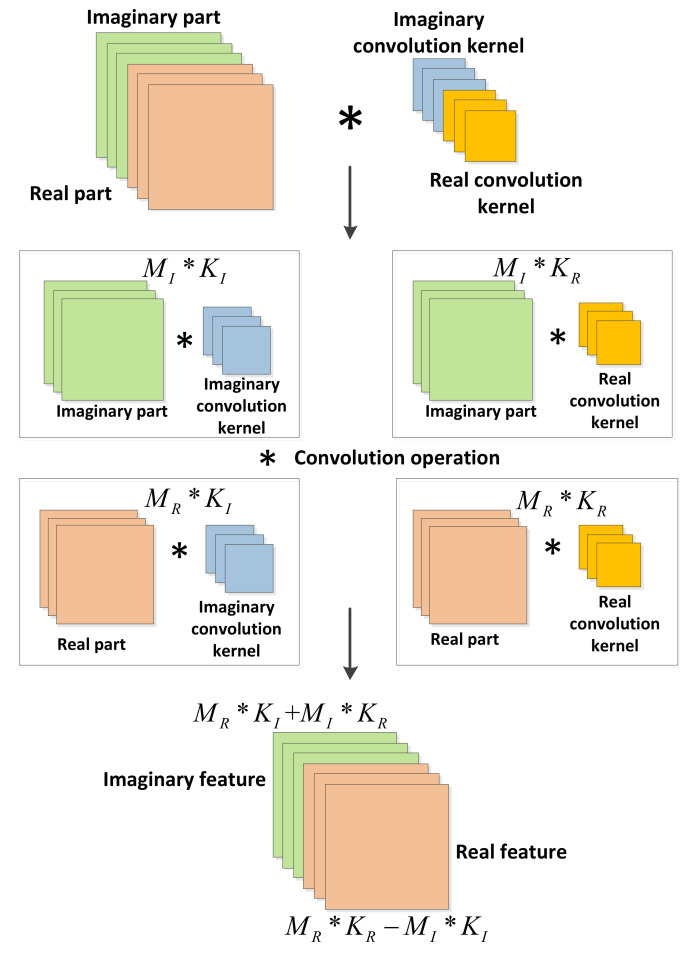
Schematic diagram of the complex-valued convolution operation.

Figure 3 shows a schematic diagram of the complex-valued convolution operation. The real and imaginary convolutions of the complex-valued signal are expressed as follows:

(7) $$\left[\begin{array}{c} Re\{M*K\}\\ Im\{M*K\} \end{array}\right]=\left[\begin{array}{cc} K_{{}_{R}} & {-K}_{I}\\ K_{I} & K_{R} \end{array}\right]*\left[\begin{array}{c} M_{R}\\ M_{I} \end{array}\right],$$

where Re{M∗K} is the real part of the signal and Im{M∗K} is the imaginary part of the signal.

The outputs of the DCN layers will carry phase information and are used as the input to the next layer.

### 3.3. Bidirectional Long Short-Term Memory Module (BiLSTM)

**Figure 4:**
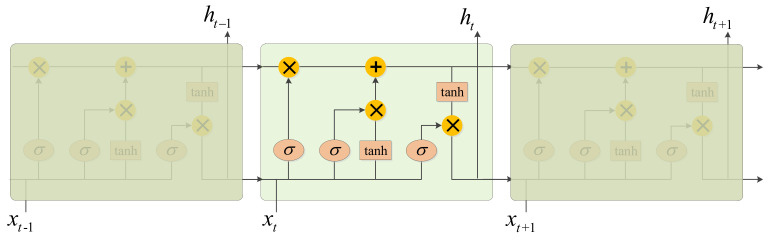
LSTM network.

**Figure 5:**
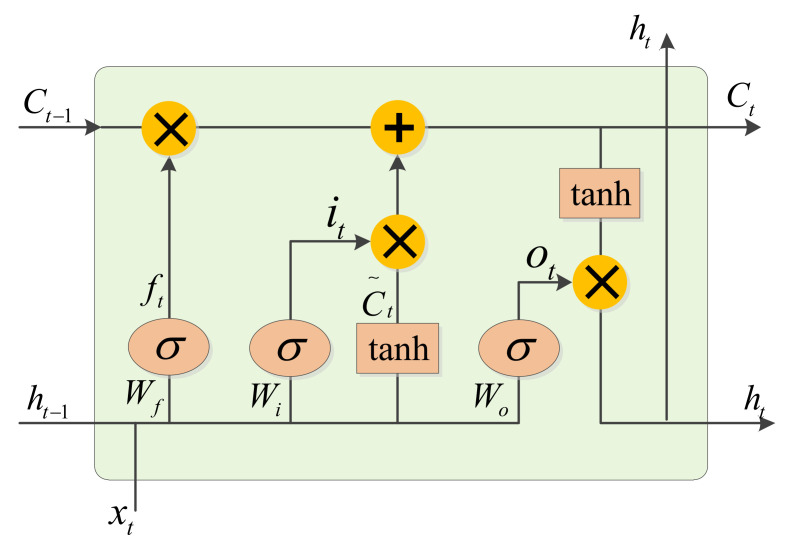
An LSTM memory cell.

We cascade a BiLSTM behind the DCN to facilitate the extraction of contextual information of features. The BiLSTM network is composed of both forward and reverse LSTM networks [26]. As shown in Figure 4, the LSTM network contains many LSTM memory cells [27] that each includes three control units, namely, an input gate, a forget gate and an output gate. Figure 5 shows a diagram of the LSTM memory cells [27].

In Figure 5, *t* represents the current moment. The input feature sequence $x_{t}$ and the output sequence of the previous time $h_{t-1}$ are input to the memory cell. The forgetting factor $f_{t}$ is obtained via the forgetting gate and is expressed as follows:

(8) $$f_{t}=\sigma \left(W_{f}\cdot \left[h_{t-1},x_{t}\right]+b_{f}\right),$$

where $W_{f}$ is the connection matrix of $x_{t}$, $h_{t-1}$. $b_{f}$ is the offset matrix, and σ is the sigmoid activation function, which is used to control the information-passing rate. The expression is

(9) $$\sigma \left(x\right)=\frac{1}{1+e^{-x}},$$

where the output value of σ is between 0 and 1. The input gate and memory status update information are

(10) $$\begin{array}{cc} \; & i_{t}=\sigma \left(W_{i}\cdot \left[h_{t-1},x_{t}\right]+b_{i}\right)\\ \; & {\tilde{C}}_{t}=\tanh\left(W_{c}\cdot \left[h_{t-1},x_{t}\right]+b_{c}\right)\\ \; & C_{t}=f_{t}*C_{t-1}+i_{t}*{\tilde{C}}_{t} \end{array}$$

where $i_{t}$ is the output of the input gate and tanh is an activation function that generates candidate values ${\tilde{C}}_{t}$. In addition, ${\tilde{C}}_{t}$ participates in the calculation to obtain the memory state $C_{t}$.

Among these various components, the memory state $C_{t}$ is the most important because it can allow information to flow through the entire link under the condition that it must remain unchanged, ensuring the integrity of the information for a long time. The output gate control factor $o_{t}$ determines whether to output information $h_{t}$ and is expressed as follows:

(11) $$\begin{array}{cc} \; & o_{t}=\sigma \left(W_{o}\cdot \left[h_{t-1},x_{t}\right]+b_{o}\right)\\ \; & h_{t}=o_{t}*\tanh\left(C_{t}\right) \end{array}$$

where $W_{o}$ is the output gate weight matrix and $b_{o}$ is the offset matrix. Compared with $C_{t}$, $h_{t}$ contains more information about the current moment. Therefore, $h_{t}$ represents short-term memory, while $C_{t}$ represents long-term memory.

**Figure 6:**
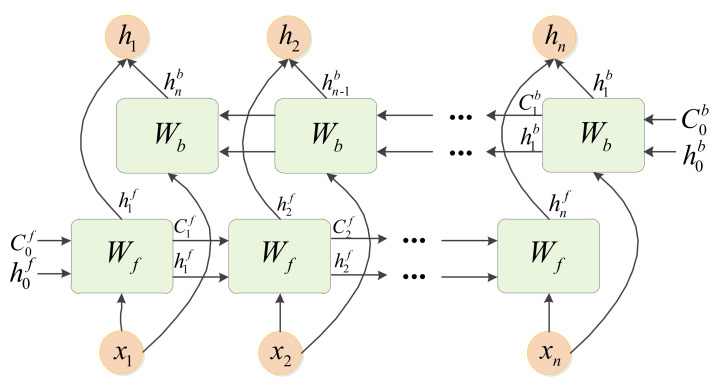
The structural operation graph of BiLSTM.

However, a classical LSTM considers only information from the previous moment. To consider both the former moment and the next moment together, the BiLSTM [26] adds reverse operations based on the LSTM model in [28,29]. Figure 6 shows a structural operation graph of the BiLSTM.

**Figure 7:**
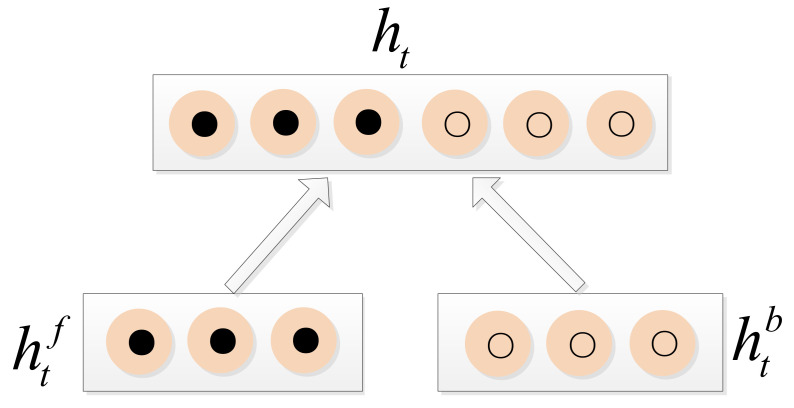
The relationship between $h_{t}$ and ${h_{t}}^{f}$, ${h_{t}}^{b}$.

As shown in Figure 6, the BiLSTM reverses the input sequence and calculates the output again in the same way as an LSTM. The final result is a stack of the forward LSTM and the reverse LSTM, which achieves the goal of considering the contextual information. The final outputs of the BiLSTM are $h_{t}$, where t=1,2,…,n, can be expressed as shown in Figure 7. The expression of $h_{t}$ is as follows:

(12) $$h_{t}=\left[h_{t}^{f},h_{t}^{b}\right].$$

The output features of the BiLSTM are mapped into a sparse space by the fully connected layer. After the network is trained, the algorithm outputs the classification probability of the corresponding modulation modes.

## 4. Experiment Results and Discussions

The relevant platform and software settings for this experiment are shown in Table 2. There are 1000 samples of each modulated signal for each SNR comprising a total of samples is 220,000 samples. The ratio of training sets to test sets is 8:2. We use the np.random.choice function to implement the proportional selection of the dataset to obtain the training sets and the test sets. In this experiment, we performed the following steps.

Step1 Initialize the DCN-BiLSTM network randomly, extract a specified number of samples in the training sets and input them into the network for training.

Step2 Compare the classification result obtained in the last layer of the network with the actual type; use the cross-entropy function to calculate the network loss value; and adjust the network weight value through the optimization algorithm.

Step3 Before the next training starts, use the loss value as a standard to measure the network performance. When it does not drop within 10 iterations, the training is stopped.

Step4 Repeat Steps 2–4 until the maximum number of training is reached or the conditions for premature termination of training are met. The maximum number of training in this article is 150. After training, the weights are saved and the classification model is output.

Step5 Input the test sets into the trained model to obtain the recognition result.

### 4.1. Algorithm Performance Comparison

**Figure 8:**
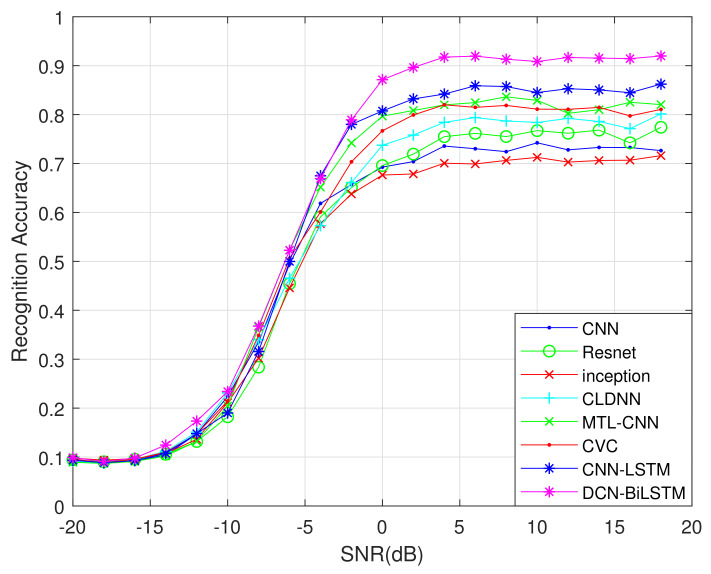
AMR rates of different networks under different signal-to-noise ratios.

We selected the recognition algorithms based on a CNN [17], Resnet [18], Inception [18], CLDNN [18], MTL-CNN [20], CVC [23] and CNN-LSTM [24] as benchmark models. A performance comparison chart for these eight recognition algorithms is shown in Figure 8.

Figure 8 shows that, from −2 to 18 dB, the accuracy of the DCN-BiLSTM network is substantially higher than the accuracy of the other seven recognition algorithms. When the SNR exceeds 4 dB, the recognition accuracy of the DCN-BiLSTM network for the 11 modulation signals can reach 90%.

**Figure 9:**
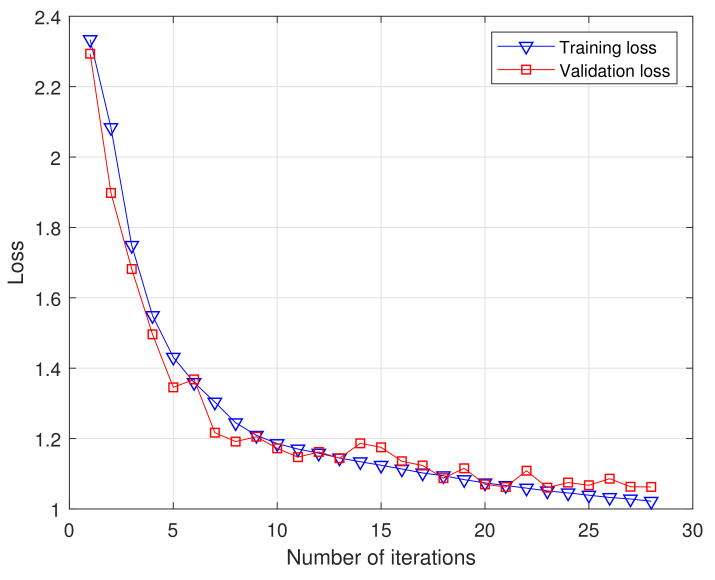
Loss value of DCN-BiLSTM algorithm.

Figure 9 shows the values of the cross-entropy loss function during DCN-BiLSTM network model training and testing. As the number of iterations increases, the training and test loss values continue to decrease, indicating that the real label and the predicted label are constantly converging. When the number of iterations reaches 23, the training and testing loss values no longer decrease, and the model obtained at this time is the best.

**Figure 10:**
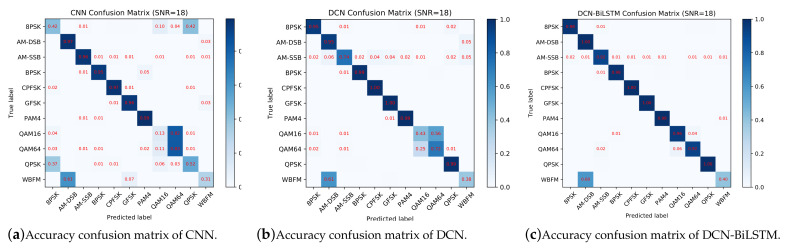
Accuracy confusion matrices of CNN, DCN and DCN-BiLSTM algorithms when SNR = 18 dB.

Figure 10 shows confusion matrices for the CNN, DCN and DCN-BiLSTM models when the SNR is 18 dB. According to Figure 10a, it is difficult for the CNN to distinguish QPSK (Quadrature Phase Shift Keying) and 8PSK (8 Phase Shift Keying) because the CNN does not fully extract the phase information: the amplitudes of QPSK and 8PSK are the same and the difference between them is the phase. In addition, 16QAM (16 Quadrature Amplitude Modulation) is often misrecognized as 64QAM (64 Quadrature Amplitude Modulation) because the constellation points of 16QAM can be found in the constellation points of 64QAM.

To fully consider the phase information, we replaced the CNN with a DCN. The accuracy confusion matrix is shown in Figure 10b. Clearly, the accuracies on the QPSK and 8PSK modulation types are much better than in Figure 10a, and the accuracy on the 16QAM and 64QAM modulation types has improved as well. However, the accuracy on the 16QAM and 64QAM types is still not good enough for practical applications; therefore, we still need to improve their recognition accuracy.

Considering the connections among data points, we cascaded the BiLSTM after the DCN to extract the contextual information of signals. An accuracy confusion matrix for the DCN-BiLSTM is shown in Figure 10c, showing that the recognition accuracy of 16QAM and 64QAM is greatly improved compared with the results in Figure 10a,b. This result indicates that the BiLSTM is useful for extracting the contextual features of signals.

As shown in Figure 10a–c, it is quite difficult to recognize wide band frequency modulation (WBFM). The reason is that the dataset uses voice signals to generate analog signals, and people’s voices have silent periods during speaking, leaving only a single carrier during the silent period. Thus, the WBFM signals can easily be misclassified as AM-DSB (amplitude modulation—double side band modulation) signals.

To fully compare the dataset recognition capabilities of the above several networks, we used an online platform that can perform statistical analysis [30]. First, we uploaded the file representing the recognition result in csv format, as shown in Table 3. Table 3 shows the recognition error rate of five types of datasets. The error rates of the first four datasets correspond to the corresponding modulated signals in the fifth dataset. According to Rodríguez-Fdez et al. [30] and the test situation in this paper, we selected Friedman [31] as the test type to be applied. We chose Holm [32], which is widely used, as the post-hoc with control method. At the same time, we set the significance level α to 0.05.

After experiments, the algorithm rankings obtained are shown in Table 4. As we can see, DCN-BiLSTM has the highest ranking and its performance is better than the other seven networks. Moreover, the ranking in Table 4 is consistent with the ranking of the recognition effect in Figure 8, which also shows the correctness of the experiment.

Table 5 summarizes the comparison between DCN-BiLSTM and the other seven algorithms by using post-hoc with control methods. By comparing p-value with α, it can be seen that the DCN-BiLSTM network is significantly different from Inception, CNN and Resnet, indicating that the proposed network has significant progress compared with them. At the same time, there are no significant differences between DCN-BiLSTM and CLDNN, CVC, CNN-LSTM and MTL-CNN, which means that the proposed algorithm inherits the excellent performance of the four networks and can replace them in the field of modulation recognition.

### 4.2. Performance Comparison When Using Different Parameters for the DCN-BiLSTM Network

To obtain the best parameter configuration for the DCN-BiLSTM network, this section studies the influences of each parameter configuration on the algorithm’s performance.

**Figure 11:**
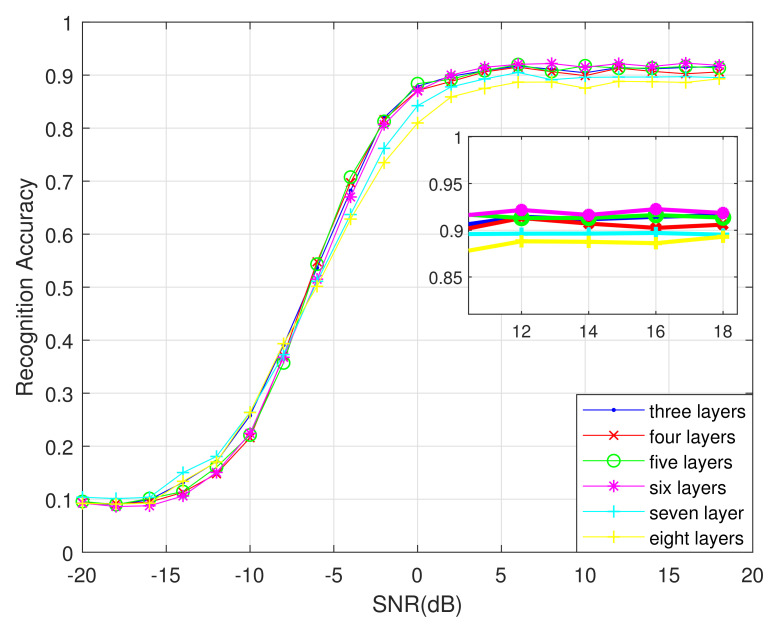
Algorithm recognition accuracy under different DCN layers.

First, we change only the number of DCN layers to find the best number of DCN layers. The different recognition results are shown in Figure 11.

As shown in Figure 11, the overall recognition rate is the highest with six DCN layers when the SNR is greater than 2 dB. With fewer than six layers, the network’s ability to extract phase features is not strong enough. With more than six layers, the network extracts redundant features, and the recognition rate no longer improves, which wastes memory. Therefore, the best number of DCN layers for this algorithm is six.

**Figure 12:**
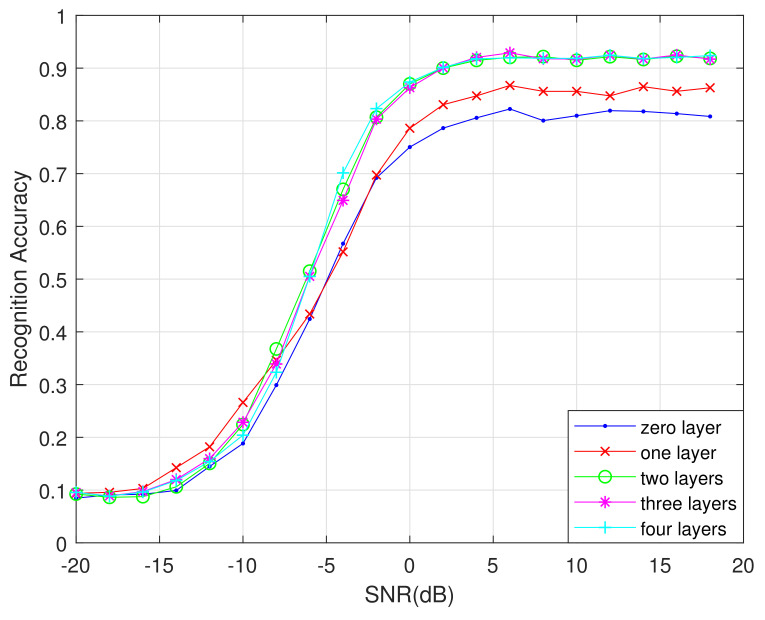
Algorithm recognition accuracy under different BiLSTM layers.

Changing the BiLSTM layer number also affects the recognition performance. Figure 12 shows a comparison of the recognition performance under different numbers of BiLSTM layers.

With fewer than two BiLSTM layers, the algorithm’s ability to process feature information is poor. With more than two layers, while the accuracy rate is equivalent to that of a two-layer BiLSTM network, the added layers cause a speed reduction and waste memory. Therefore, it is best to set the number of BiLSTM layers to two.

Based on the previous analysis, the final DCN-BiLSTM network parameters are shown in Table 6.

### 4.3. Five-Fold Cross Validation

**Figure 13:**
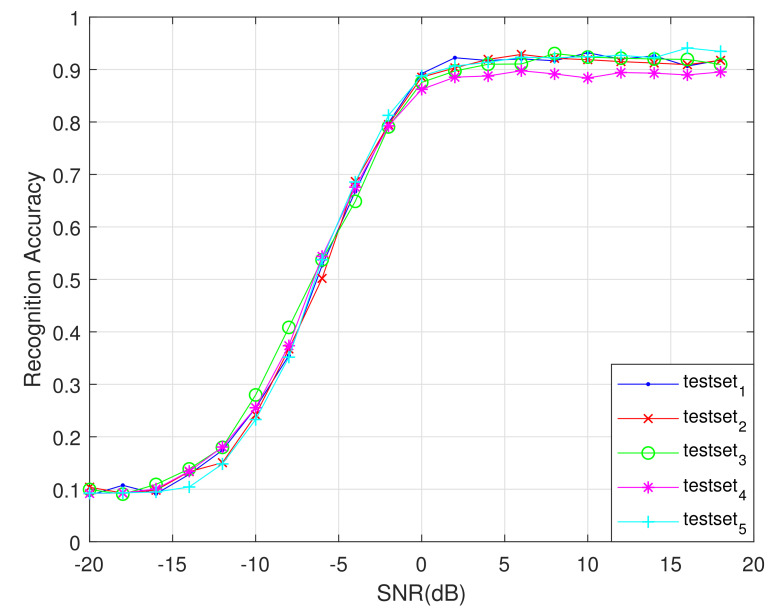
Recognition rate curve for five-fold cross-validation.

**Figure 14:**
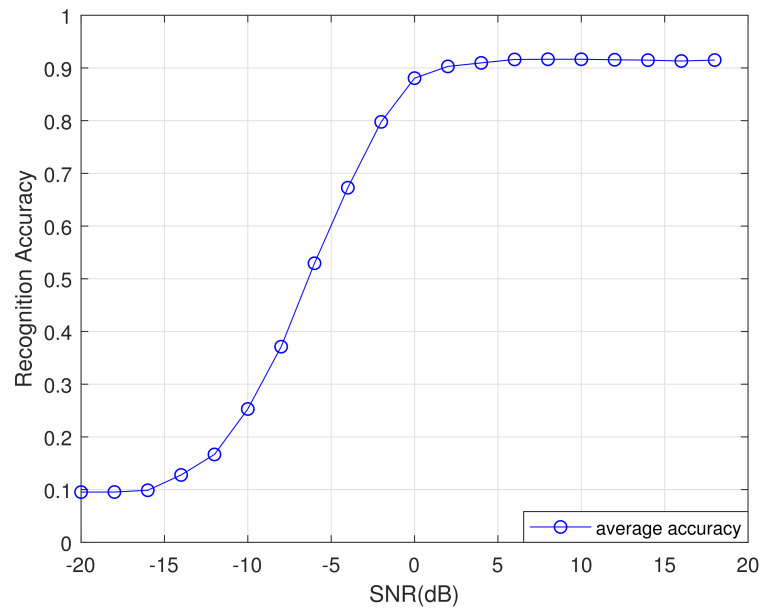
Average recognition rate curve of five-fold cross-validation.

To evaluate the performance of the network proposed in this article, we used the five-fold cross-validation method to train and test the network in this part. We divide the dataset into five parts equally, and use one part as the test sets each time and the remaining four parts as the training sets. Finally we obtained five training and test results, as shown in Figure 13. The average recognition rate curve of these five times is shown in Figure 14. As shown in Figure 13 and Figure 14, when SNR is greater than 4 dB, the recognition accuracy of the proposed network is more than 90% under five trainings, covering the entire dataset, which illustrates the rationality and stability of the designed network.

## 5. Conclusions

In this paper, we propose a classification algorithm based on the DCN-BiLSTM network that achieves direct recognition of 11 different types of modulated signals. First, DCN layers are used to extract the phase features of the modulation signal. Then, BiLSTM layers are used to extract the contextual information and construct a bidirectional long short-term memory model for the features. Compared with previous network recognition algorithms based on CNN, Inception, Resnet, CLDNN, MTL-CNN, CVC and CNN-LSTM, the recognition accuracy of the DCN-BiLSTM network is significantly higher under high SNR. However, even when the SNR is as low as 4 dB, the recognition accuracy rate of the DCN-BiLSTM network can still reach 90%.

However, the DCN-BiLSTM network has a slow training speed, and the method works satisfactorily only for signals whose frequency offset and sampling frequency offset are within a certain range. In addition, the identified signal must belong to one of the 11 specified types. In future work, we plan to modify or optimize these issues. In particular, on the one hand, for the training speed issue, we can use other GPUs with stronger computing capabilities to speed up training. On the other hand, in terms of network structure, each LSTM unit in the BiLSTM layers contains three gate functions, resulting in more parameters, which is the main reason for the slow network training speed. Therefore, we can try to optimize the LSTM unit to reduce the number of parameters and increase the training speed, such as reducing or simplifying the gate function.

In addition to the phase information and contextual information mentioned in this paper, many other characteristics of modulated signals could be considered, such as time–frequency domain characteristics and constellation characteristics. In future work, we plan to use other features in combination with the DCN-BiLSTM network to improve the modulation signal identification performance.

## Figures and Tables

**Table 1:** The specific dataset parameters.

Data Source	RML2016.10a
Modulation types	AM-DSB, AM-SSB, WBFM, BPSK, 8PSK, QPSK, CPFSK,
	GFSK, PAM4, 16QAM, 64QAM
Data length	128
Data dimension	2 × 128
Sampling frequency	200 kHz
Sampling rate offset standard deviation	0.01 Hz
Maximum sampling rate offset	50 Hz
Carrier frequency offset standard deviation	0.01 Hz
Maximum carrier rate offset	500 Hz
Number of sinusoids used in frequency selective fading	8
Maximum doppler frequency used in fading	1
Fading model	Rician
Rician K-factor	4
Fractional sample delays for the power delay profile	[0.0, 0.9, 1.7]
Magnitudes corresponding to each delay time	[1, 0.8, 0.3]
Filter length to interpolate the power delay profile	8
Standard deviation of the AWGN process	$\sqrt{10^{-\frac{SNR}{10}}}$
SNR(dB)	−20:2:18

**Table 2:** The relevant experimental platform and software settings.

Signal generation software platform	GNU Radio
Deep learning simulation platform	TensorFlow 1.7.0
Deep learning library	Keras
Hardware acceleration platform	NVIDIA GTX1080Ti

**Table 3:** The recognition error rate of different networks for different datasets.

Datasets	DCN-BiLSTM	CNN	Resnet	Inception	CLDNN	MTL-CNN	CVC	CNN-LSTM
8PSK	0.442	0.632	0.514	0.574	0.523	0.483	0.461	0.498
16QAM	0.422	0.876	0.758	0.794	0.773	0.621	0.826	0.78
64QAM	0.32	0.381	0.517	0.553	0.474	0.614	0.425	0.506
QPSK	0.429	0.783	0.698	0.722	0.601	0.518	0.583	0.499
RML2016.10a	0.379	0.492	0.476	0.498	0.458	0.445	0.453	0.445

**Table 4:** Algorithm rankings.

Rank	Algorithm
1.00000	DCN-BiLSTM
3.70000	CNN-LSTM
3.70000	MTL-CNN
4.00000	CVC
4.80000	CLDNN
5.20000	Resnet
6.60000	CNN
7.00000	Inception

**Table 5:** The comparison of DCN-BiLSTM with other algorithms using the post-hoc with control methods.

Comparison	Statistic	Adjusted *p*-Value	Result
DCN-BiLSTM vs Inception	3.87298	0.00075	H0 is rejected
DCN-BiLSTM vs CNN	3.61478	0.00180	H0 is rejected
DCN-BiLSTM vs Resnet	2.71109	0.03353	H0 is rejected
DCN-BiLSTM vs CLDNN	2.45289	0.05669	H0 is accepted
DCN-BiLSTM vs CVC	1.93649	0.15842	H0 is accepted
DCN-BiLSTM vs CNN-LSTM	1.74284	0.16272	H0 is accepted
DCN-BiLSTM vs MTL-CNN	1.74284	0.16272	H0 is accepted

**Table 6:** The DCN-BiLSTM network parameters.

Layer	Activation Function	Output Dimensions
Input	/	(128, 2)
DCN layer L1	ReLU	(None, 64, 32)
DCN layer L2	ReLU	(None, 64, 128)
DCN layer L3	ReLU	(None, 64, 128)
DCN layer L4	ReLU	(None, 64, 128)
DCN layer L5	ReLU	(None, 64, 128)
DCN layer L6	ReLU	(None, 64, 256)
BiLSTM layer B1	/	(None, 64, 512)
BiLSTM layer B2	/	(None, 256)
Dense	Softmax	(None, 11)

## Data Availability

Not applicable.

## Abbreviations

The following abbreviations are used in this manuscript:

AMR	Automatic modulation recognition
DCN-BiLSTM	Deep complex network-bidirectional long short-term memory network
DCN	Deep complex network
BiLSTM	Bidirectional long short-term memory
CNN	Convolutional neural network
SNR	Signal-to-noise ratio
ML	Maximum likelihood
FB	Feature-based
SVMs	Support vector machines
ANNs	Artificial neural networks
DL	Deep learning
QAM	Quadrature Amplitude Modulation
STN	Spatial transformation network
KCRDP	Kernel collaborative representation and discriminative projection
Resnet	Residual network
CLDNN	Long short-term memory deep neural network
MTL-CNN	CNN that uses a multitask learning scheme
CM	Correction module
DNN	Deep neural network
CVCs	Complex convolutions
LSTM	Long short-term memory
ReLU	Rectified linear unit
AM-DSB	Amplitude modulation-Double sideband modulation
AM-SSB	Amplitude modulation-Single sideband modulation
WBFM	Wide band frequency modulation
BPSK	Binary Phase Shift Keying
8PSK	8 Phase Shift Keying
QPSK	Quadrature Phase Shift Keying
CPFSK	Continuous Phase Frequency Shift Keying
GFSK	Gauss Frequency Shift Keying
PAM4	Pulse Amplitude Modulation 4
16QAM	16 Quadrature Amplitude Modulation
64QAM	64 Quadrature Amplitude Modulation
GPU	Graphic Processing Unit
